# Newspaper Medicine

**Published:** 1906-12

**Authors:** 


### EDITORIAL NOTES AND COMMENTS.

The Business office of THE JOURNAL-RECORD is 1007-1008 Century Building.

The Editorial office is 318-319-320 The Prudential.

Address all Business communications to Dr. M. B. Hutchins, Manager.

Make remittances payable to THE ATLANTA JOURNAL-RECORD OF MEDICINE.

On matters pertaining to the Editorial and Original Communications address Dr. Bernard Wolff, Atlanta.

Reprints of original articles will be furnished at cost price. Requests for the same should always be made *on the manuscript.*

We will present, post-paid, on request, to each contributor of an original article, twenty (20) marked copies of THE JOURNAL-RECORD containing such article.

### NEWSPAPER MEDICINE.

NEWSPAPER misconceptions of medical matters are frequently grotesquely amusing. At times the press will startle the world with the announcement of some wonderful medical discovery, a tuberculosis or cancer cure, perhaps, which is destined to revolutionize the treatment of these diseases. It is not at all unlikely that these startling discoveries have been consigned by doctors to the rubbish heap months or years before.

Newspaper surgical operations are prone to be of a "delicate" nature, and a "successful" operation means one in which the patient comes off the operating-table alive. The fact that the success of an operation is rarely ever at once established is entirely overlooked.

A case in point of the flossy style of dealing with pathology by the newspapers is that furnished by the discovery in the recruiting-office of the Marine Corps here of a man presenting the condition known as dermographism. The article describing the case was entitled "Devil's Writing." The officer in charge of the recruiting-office and the surgeon of the Marine Corps were alleged to have invited medical men, skin specialists and skin experts (whoever and whatever these may be) and representatives of the press to witness the singular phenomenon of writing on a man's skin. The surgeon is charged with making the following running comment on the case: "It is a urticaria case," said Surgeon Hart, never looking up from his study of the body. "I've heard of it before, but—turn this way, please. Now, there. Still, please; don't move. Yes, ding it, that's what it is. I've been in the ser—"

"What you call it, doctor?" asked the recruit, a tremor in his voice, a paleness about his lips. "Wha—"

"Urticaria, the devil's writing. I've heard of it and read of it often; but—but this is my first actual contact with it. I've"—and so forth and so on.

Urticaria, "a word as old as the medical profession itself," is defined by a "a desk dictionary" as "the outer or upper cuticle—the epidermis." The definition makes quite clear the significance of devil's writing and also the surgeon's inexperience with it, for when the epidermis becomes the cuticle and urticaria becomes the epidermis, in the mix-up many things may come to light that were hitherto obscure.

The article describes with much detail the devil's writing, with many cracks of pure humor and playful quips and droll side-thrusts which go to make up an unusually rich exhibit of journalistic slush, fudge and rot.

"Devil's writing," as applied to dermographism, is quite as interesting as the definition of urticaria, and quite as new. Sufferers from urticaria will agree that the devil has a hand in it, and will welcome so neatly descriptive a term, but it is a novelty in nomenclature nevertheless.

The unfortunate surgeon who was a victim of reportorial misconception of medical terms deserves the heartfelt sympathy of the profession. It is nearly as bad as having to suffer from typographical errors and almost as irremediable.

Instances like the foregoing lead to the conclusion that newspapers should either let medical affairs alone or employ reporters who have enough familiarity with medicine and medical terminology to enable them to write with some intelligence. There is enough in medicine to satisfy the yellow taste of seekers after sensation, and if such things have to be given out, let it be with some reference to facts and actualities.

